# Proportion and Risk Factors of Silent Vertebral Fractures Among Egyptian Females With Fragility Hip Fracture Presenting to the Emergency Room of Ain Shams University Hospitals

**DOI:** 10.7759/cureus.46214

**Published:** 2023-09-29

**Authors:** Mohamed A Abdelrahman Ali, Hala s Sweed, Mohamed F Allam, Walaa W Aly, Abeer H Mohamed Matter, Walid E Abdelalim Elshabrawy

**Affiliations:** 1 Geriatrics, Ain Shams University, Cairo, EGY; 2 Family Medicine, Ain Shams University, Cairo, EGY; 3 Orthopedic Surgery, Ain Shams University, Cairo, EGY

**Keywords:** elderly, vertebral fracture, osteoporosis, hip fracture, fragility fracture

## Abstract

Background

Fragility fractures caused by osteoporosis are known to increase the risk of further fragility fractures. Also, several factors have been associated with an increasing risk of fracture in postmenopausal women with osteoporosis, as prior fracture, advancing age, low bone mineral density (BMD), greater risk or history of falls certain, pharmacologic therapies such as glucocorticoids, and medical conditions increase the risk of secondary osteoporosis and related fractures through their direct impact on bone density or structure. Menstrual history including age at menarche menopause and a history of amenorrhea is documented as a predicting osteoporotic fracture.

Objective

The aim of the current study is to find the proportion of hidden vertebral fractures among Egyptian females with fragility hip fracture.

Patients and methods

A cross-sectional study was conducted on patients who presented to the orthopedic emergency room (ER) of Ain Shams University Hospitals in Cairo, Egypt, from September 2020 to September 2021. Our inclusion criteria include females aged 40 years or older, who presented to the ER with fragility hip fractures. A simple random sample of females fulfilling our inclusion criteria for osteoporotic hip fracture was thoroughly investigated. Conventional lateral and anteroposterior radiographs of the dorsolumbar spine were obtained excluding those with high-impact fractures or pathological fractures.

Results

During the study period, a total of 43,935 persons presented to the orthopedic ER, of whom 30,901 were females, comprising 70.03% of total orthopedic ER visits. A sample of 150 females met our inclusion criteria. Results showed that 16 of our 150 cases had concomitant vertebral fracture, meaning that 10.7% of cases had hidden vertebral fracture at the time of osteoporotic hip fracture, as diagnosed by the screening lumbosacral plain X-rays. Older age at menarche, younger age at menopause, and amenorrhea are shown to be risk factors for hidden vertebral fracture in Egyptian females.

Conclusion

Osteoporosis is a complex and costly disease. Osteoporotic fractures may be largely preventable, as environmental factors are open to intervention, and effective pharmacological agents are available. Concomitant hidden vertebral fracture is prevalent among females with osteoporotic hip fractures, and those who had later menarche, earlier menopause, and menstrual irregularities have a higher incidence of developing associated vertebral fracture, which warrants identification and management to evade complications and mortality.

## Introduction

The prevalence of hip fractures in the elderly population is rising as population around the world get older. In fact, 6.3 million hip fractures among the elderly are predicted to occur in 2050 [1]. In Egypt, the prevalence rate of osteoporosis was 28.4% in females and 21.9% in males [2]. Additionally, osteopenia affected 53.9% of females and 26% of males in Upper Egypt's rural areas; the frequency of osteoporosis in postmenopausal women was greater at 47.8% [3].

As regards vertebral fracture, for both sexes, a 2012 study in Norway showed that the prevalence increases with age, ranging from 3% in female participants below 60 years of age (7.5% in men) to 19% in female participants over 70 years of age (20% in men) [4].

Hip fractures of femoral neck fractures and inter-trochanteric fractures in patients ≥65 years of age are usually due to low-energy trauma (such as a fall from a standing position). These fractures are known as “osteoporotic fractures” and are characterized by reduced bone mass and microstructural destruction [5]. Poor physical condition, increased underlying diseases, and comorbidity are likely to cause decreasing bone strength and corresponding increases in fracture risk [6].

An increased risk of fracture in postmenopausal women with osteoporosis has been linked to a number of conditions, and a major risk factor for subsequent fractures is a prior fracture [7]. Ageing, low bone mineral density (BMD), a higher risk of falling or a history of falling, use of certain pharmacologic treatments such as glucocorticoids, and certain medical conditions all increase the risk of secondary osteoporosis and related fractures by having an immediate effect on bone density or structure. In addition, specific conditions and medications increase fracture risk indirectly through mobility and/or physical, cognitive, visual, and sensory deficits that increase the risk of falls [8].

Hip fractures are frequent and disabling incidents that result in severe limitations to the person and often serve as a turning point in a downward cycle leading to death [9]. Hip fractures in the elderly are associated with increased morbidity and mortality, and the risk of hip fracture is high and ranges from 40% to 50% in women and 13% to 22% in men [10], resulting in a health and socioeconomic burden [11].

Osteoporosis is typically seen as a "woman's illness" since postmenopausal women have a considerably higher prevalence of the condition and risk of fracture than older males; 60-year-old women have a roughly 44% lifetime risk of fracture, which is more than twice as high as the 25% risk for men in the same age group [12]. Some studies have reported overall one-year mortality rates between 18% and 31%, with men generally having a higher mortality rate than women [13]. In addition, hip fractures cause patients' excessive pain, depression, and postoperative anxiety [14].

Costs associated with hospitalization, surgery, and rehabilitation for individuals with these fractures are disturbingly high across the globe for healthcare systems [15]. According to a study by Iorio et al. [16], the calculated cost for treating hip fractures surgically ranged from $20,000 to $24,000 per case, depending on whether internal fixation, hemiarthroplasty, or total hip arthroplasty was used; this is because hip fracture surgery requires expensive implants.

While hip fractures remain a key driver of osteoporosis-related costs, some research indicates that osteoporosis-related, non-hip fractures, such as those of the spine, wrist/forearm, pelvis, humerus, and leg, are more prevalent than hip fractures. It was reported that of the 2 million osteoporotic fractures estimated in 2005, hip fractures account for only 14% and spine fractures represent 27% [17].

The total cost of vertebral fractures in the European Union (EU) was projected to be €377 million per year. Across the EU, the hospital cost of a vertebral fracture was on average 63% of that of a hip fracture [18].

The objective of the current study is to find the proportion and risk factors of hidden vertebral fractures among Egyptian females more than 40 years of age with fragility hip fracture who presented to the orthopedic emergency room (ER) in Ain Shams University Hospitals (ASUHs).

## Materials and methods

Patients seen in the orthopedic ER of ASUHs in Cairo, Egypt, were included in this cross-sectional study. Participants were recruited over the course of a year (from September 2020 to September 2021) at the ASUH, a tertiary referral facility that is well equipped to treat trauma patients.

 The inclusion criterion in this study was patients (females) older than 40 years old presenting with fragility hip fractures, while the exclusion criterion was patients who did not fulfill the inclusion criteria mentioned earlier.

Sampling method was used employing consecutive sampling. A simple random sample of females fulfilling our inclusion criterion for osteoporotic hip fracture was thoroughly investigated.

The estimated sample size was calculated according to the records of ASUHs and the registered numbers of patients who visited the ER in the previous years. The level of confidence was 95%, and the level of precision was 3%. The percentage of females older than 40 years with a hip fracture was 2.7%. The provisional sample size was 111. The percentage of expected lost follow-up patients or patients with incomplete data was 15%, and the final sample size was 131 (approximated to 150). During the study period, we recruited 150 female patients fulfilling our inclusion criterion.

All participants were subjected to comprehensive assessment. History was taken from the patients admitted to ASUHs’ orthopedic ER and confirmed by a family member or caregiver to obtain an accurate history. Risk factors for fragility fractures included age, sex, weight in kilograms, height in centimeters, history of previous fragility fracture, history of parent hip fracture, current smoking, intake of glucocorticoids, history of rheumatoid arthritis, history of secondary osteoporosis (this included patients who have a disorder strongly associated with osteoporosis, such as type I [insulin dependent] diabetes, osteogenesis imperfecta in adults, untreated long-standing hyperthyroidism, hypogonadism, chronic malnutrition, malabsorptive syndromes, and chronic liver disease), intake of alcohol (3 or more units/day), and menstrual history (age at menarche, age at menopause, regularity of menstrual cycles, duration of menstrual period in days, and history of amenorrhea or metrorrhagia). General examination included vital data (blood pressure, pulse, complexion, level of edema if present), and local examination included full system-specific examination according to the cause of admission. Anthropometric data were collected including body mass index (BMI); weight and height were measured according to the Frisancho technique [19]. By using an approximation formula based on simple anthropometric measurements (body height, and waist and hip circumference), it was possible to obtain a quick and accurate approximation of body weight (women: height in cm − 100) − (height in cm − 150)/2) [20]. Height was measured with a ruler with the patient in upright standing position without shoes. Height was recorded to the nearest meter. Standing height measurement in older people was difficult to obtain, and impossible or inaccurate in some situations, because of an inability to stand straight or steadily due to pain, weakness, disability, or spinal deformities such as kyphosis or due to osteoporosis or any other factors, and thus it can be calculated using the equation: height = 53.781+ 2.131 (knee height) [21]. Hip fractures were identified by plain film radiographs with an anteroposterior view of the pelvis along with a lateral view. Conventional lateral and anteroposterior X-ray radiographs of the dorsolumbar spine were obtained for patients presenting with hip fractures to screen for concomitant hidden vertebral fracture as interpreted and diagnosed by a radiology specialist, where a vertebral deformity in T4-L4 of more than 20% of loss in height with a reduction in area of more than 10-20% was defined as a fracture.

The Ain Shams University Ethical Committee granted their approval (FAMSU M D 418/2019). A written informed consent was obtained from every participant. This work has been carried out in accordance with the Code of Ethics of the World Medical Association (Declaration of Helsinki) for studies involving humans.

Data were collected, revised, coded, and entered into the Statistical Package for Social Sciences (SPSS) Version 22.0 (IBM Corp., Armonk, NY, USA). The quantitative data were presented as means, standard deviations, and ranges when parametric, and as medians and interquartile ranges (IQR) when non-parametric. Also, qualitative variables were presented as numbers and percentages. Inferential analyses were conducted for quantitative variables using the independent t-test in cases of two independent groups with normally distributed data, the paired t-test in cases of two dependent groups with normally distributed data, and the Mann-Whitney U in cases of two independent groups with non-normally distributed data. In qualitative data, inferential analyses for independent variables were conducted using the chi-square test for differences between proportions and Fisher’s exact test for variables with small, expected numbers. The adjusted risk factors for hidden vertebral fracture were obtained using the logistic regression analysis. The dependent variable was the presence and absence of hidden vertebral fracture in all patients with hip osteoporotic fracture. All variables described previously were considered as possible candidates for the final model. The initial multivariable model construction consisted of the preliminary selection of variables using a manual purposeful selection method and a relatively large significance level (alpha approximately 0.25). Subsequently, the resulting model was reduced using a likelihood ratio test with a significance level of 0.05 [22].

## Results

During the study period, 43,935 patients presented to the orthopedic ER of ASUHs. Of them, 30,901 (70.03%) patients were females, of whom 945 (3.05%) had hip fractures, and thus 150 participants were included in the study as they met our study inclusion criterion (Table 1).

**Table 1:** Frequency of osteoporotic hip fractures in females presented to the ER. ER, emergency room

Total orthopedic ER cases	N = 43,935	
	N	%
Female	30,901	70.03
hip fracture in females	945	3.05

Table 2 shows the difference between the social data and menstrual history in the fracture groups where it was further subdivided into two groups. The first group comprised patients with hip fracture and the second group comprised patients with hip fracture and concomitant vertebral fractures, where the comparison showed statistical significance between the two groups in menarche age (p=0.010), menopause age (p=0.011), amenorrhea (p=0.003), and metrorrhagia (p=0.035).

**Table 2:** Socioeconomic data and menstrual history in patient groups with hip fracture. Note: p-value > 0.05 is NS; p-value < 0.05 is S, and p-value < 0.01 is HS. Low education means less than six years of formal education, middle education means six years or more of education but not a university graduate, and high education means completing a university degree or equivalent *Independent t-test. **Chi-square test. NS, non-significant; S, significant; HS, highly significant; SD, standard deviation

Variable		Patients (N = 150)	Vertebral fracture		Test value	P-value	Sig.
			No	Yes			
			N = 134	N = 16			
Age	Mean ± SD	63.96 ± 6.37	63.73 ± 6.18	65.88 ± 7.74	-1.275*	0.204	NS
	Range	50–84	50–84	53–81			
Education	Low education	54 (36.0%)	48 (35.8%)	6 (37.5%)	0.137**	0.934	NS
	Middle education	53 (35.3%)	48 (35.8%)	5 (31.3%)			
	High education	43 (28.7%)	38 (28.4%)	5 (31.3%)			
Marital status	Single	64 (42.7%)	59 (44.0%)	5 (31.3%)	0.954**	0.329	NS
	Married	86 (57.3%)	75 (56.0%)	11 (68.8%)			
History of previous fragility fracture	Yes	38 (25.3%)	35 (26.1%)	3 (18.8%)	0.410**	0.522	NS
	No	112 (74.4%)	99 (73.9%)	13 (81.3%)			
Current smoking	Non-smoker	63.96 ± 6.37	124 (92.5%)	15 (93.8%)	0.031**	0.860	NS
	Smoker	50–84	10 (7.5%)	1 (6.3%)			
Menarche age	Mean ± SD	14.72 ± 1.09	14.64 ± 1.11	15.38 ± 0.62	-2.597*	0.010	S
	Range	10–16	10–16	14–16			
Menopause age	Mean ± SD	49.35 ± 1.41	49.45 ± 1.40	48.50 ± 1.26	2.591*	0.011	S
	Range	47–52	47–52	47–51			
Years of menstruation	Mean ± SD	34.63 ± 1.73	41.64 ± 2.37	34.63 ± 1.73	-0.760*	0.448	NS
	Range	31–39	36–47	31–39			
Regularity of menstrual cycles	Irregular	37 (24.7%)	35 (26.1%)	2 (12.5%)	1.427**	0.232	NS
	Regular	113 (75.3%)	99 (73.9%)	14 (87.5%)			
Menorrhagia	No	138 (92.0%)	122 (91.0%)	16 (100.0%)	1.557**	0.212	NS
	Yes	12 (8.0%)	12 (9.0%)	0 (0.0%)			
Amenorrhea	No	112 (74.7%)	105 (78.4%)	7 (43.8%)	9.051**	0.003	HS
	Yes	38 (25.3%)	29 (21.6%)	9 (56.3%)			
Metrorrhagia	No	129 (86.0%)	118 (88.1%)	11 (68.8%)	4.426**	0.035	S
	Yes	21 (14.0%)	16 (11.9%)	5 (31.3%)			
Menstruation period	3–4 days	75 (50.0%)	66 (49.3%)	9 (56.3%)	1.883**	0.597	NS
	5–7 days	49 (32.7%)	43 (32.1%)	6 (37.5%)			
	8–10 days	12 (8.0%)	12 (9.0%)	0 (0.0%)			
	>10 days	14 (9.3%)	13 (9.7%)	1 (6.3%)			
Weight (in kg)	Mean ± SD	80.64 ± 9.11	80.63 ± 9.10	80.69 ± 9.46	-0.022*	0.982	NS
	Range	61–110	61–110	70–98			
Height (in meters)	Mean ± SD	1.78 ± 0.06	1.78 ± 0.06	1.79 ± 0.05	-0.533*	0.595	NS
	Range	1.51–1.88	1.51–1.88	1.71–1.87			
Body mass index	Mean ± SD	25.33 ± 2.83	25.35 ± 2.81	25.16 ± 3.13	0.242*	0.809	NS
	Range	20.24–34.21	20.24–34.21	21.91–30.44			

Table 3 shows that in the study, chronic comorbidities (diabetes mellitus, hypertension, ischemic heart disease, cerebrovascular stroke, congestive heart failure, atrial fibrillation, dementia, rheumatoid arthritis, chronic kidney diseases, chronic liver disease, thyroid disease, parathyroid disease, number of chronic conditions, intake of steroids, and intake of antiepileptics) showed no statistical significance between the two groups of the study.

**Table 3:** Chronic comorbid conditions in the studied group. Note: p-value > 0.05 is NS, p-value < 0.05 is S, and p-value < 0.01 is HS. *Chi-square test. ‡Mann–Whitney test NS, non-significant; S, significant; HS, highly significant; IQR, interquartile range

Variable		Patient group	Vertebral fracture		Test value	P-value	Sig.
			No	Yes			
		N = 150	N = 134	N = 16			
Diabetes mellitus		56 (37.3%)	50 (37.3%)	6 (37.5%)	0.000*	0.988	NS
Hypertension		52 (34.7%)	45 (33.6%)	7 (43.8%)	0.652*	0.419	NS
Ischemic heart disease		16 (10.7%)	13 (9.7%)	3 (18.8%)	1.228*	0.268	NS
Cerebrovascular stroke		4 (2.7%)	4 (3.0%)	0 (0.0%)	0.491*	0.484	NS
Congestive heart failure		7 (4.7%)	5 (3.7%)	2 (12.5%)	2.470*	0.116	NS
Atrial fibrillation		6 (4.0%)	6 (4.5%)	0 (0.0%)	0.746*	0.388	NS
Dementia		23 (15.3%)	21 (15.7%)	2 (12.5%)	0.111*	0.739	NS
Rheumatoid arthritis		8 (5.3%)	8 (6.0%)	0 (0.0%)	1.009*	0.315	NS
Chronic kidney diseases		32 (21.3%)	28 (20.9%)	4 (25.0%)	0.143*	0.705	NS
Chronic liver disease		21 (14.0%)	20 (14.9%)	1 (6.3%)	0.893*	0.345	NS
Thyroid disease		150 (100.0%)	5 (3.7%)	0 (0.0%)	0.618*	0.432	NS
Parathyroid disease		5 (3.3%)	8 (6.0%)	0 (0.0%)	1.009*	0.315	NS
Number of chronic conditions	Median (IQR)	1 (0.0–2.0)	2 (2–3)	2 (2–3)	-0.270‡	0.787	NS
	Range	0–5	1–6	1–6			
Steroids intake	No	130 (87.7%)	117 (87.3%)	13 (81.3%)	0.455*	0.500	NS
	Yes	20 (13.3%)	17 (12.7%)	3 (18.8%)			
Antiepileptics intake	No	137 (91.3%)	123 (91.8%)	14 (87.5%)	0.332*	0.564	NS
	Yes	13 (8.7%)	11 (8.2%)	2 (12.5%)			

As shown in Table 4, the univariate regression analysis for predictors of vertebral fractures highlighted that significant predictors were amenorrhea (p=0.005) and metrorrhagia (p=0.044), while the multivariate regression analysis demonstrated that the still significant predictors were menopause age ≤ 49 (p=0.020) and amenorrhea (p=0.022).

**Table 4:** Logistic regression analysis for predictors of hidden vertebral fracture. Note: p-value > 0.05 is NS, p-value < 0.05 is significant, and p-value < 0.01 is HS. NS, non-significant; S, significant; HS, highly significant; CI, confidence interval; OR, odds ratio

Variable	Univariate				Multivariate			
	P-value	OR	95% CI for OR		P-value	OR	95% CI for OR	
			Lower	Upper			Lower	Upper
Menarche age	0.058	7.333	0.938	57.313	0.065	7.279	0.886	59.826
Menopause age	0.052	3.621	0.986	13.295	0.020	5.152	1.297	20.463
Amenorrhea	0.005	4.655	1.597	13.570	0.022	3.926	1.216	12.671
Metrorrhagia	0.044	3.352	1.031	10.899	0.096	3.258	0.810	13.103

## Discussion

Hip fracture is a significant public health issue that is linked to high rates of morbidity and mortality in both developed and developing nations. They are significant contributors to elderly hospital admissions and a significant public health concern with wide-ranging effects on people's personal, social, and economic well-being in the Western societies [23].

Women begin to experience bone loss between 30 and 39 years of age, and their risk of fractures increases significantly after menopause. Globally, one in three women and one in five men over the age of 50 will suffer an osteoporotic fracture, with hip fractures being the most severe type of fracture [24].

Gender is a crucial factor in many cases, concerning both biological and social aspects, but little is known about how gender affects falls and subsequent hip fractures [25]. The situation of Egyptian women, specific hip fracture incidence rates and resulting morbidity and mortality, and the magnitude of the problem in general remain unclear due to gaps in studies, information, and health system procedures.

The present study showed that in the studied group, 16 (10.7%) patients had vertebral fractures. This is in agreement with what was previously reported in the study [26], which showed that 33.3% of patients had associated vertebral fracture with the hip fracture while being presenting to emergency services. The presence of a vertebral fracture is a risk factor for subsequent fracture at any given BMD. In recent years, vertebral fracture assessment has become the established method in clinical use, providing supplemental information on bone strength and future fracture risk [27].

During the study, participants’ past medical history was gathered, and comparing the two groups regarding the chronic comorbidities (diabetes mellitus, hypertension, ischemic heart disease, cerebrovascular stroke, congestive heart failure, atrial fibrillation, dementia, rheumatoid arthritis, chronic kidney diseases, chronic liver disease, thyroid disease, parathyroid disease, number of chronic conditions, intake of steroids, and intake of antiepileptics), we found no statistical significance. While no studies assessed these factors in relation to vertebral fractures, a tremendous amount of research studied these factors in relation to osteoporosis and osteoporotic fracture in general; for instance, the two most prevalent conditions in the study were hypertension and diabetes mellitus (37.3% and 34.7%, respectively). In the meta-analysis by Li et al. [28], which included 1,430,431 participants, and 148,048 osteoporotic fracture cases, hypertension was shown to be strongly related to osteoporosis and osteoporotic fracture, and the risk of osteoporotic fracture among individuals with hypertension was higher (p < 0.001) than that among individuals without hypertension. Regarding diabetes mellitus, a study by Yokomoto-Umakoshi et al. [29] compared various parameters between patients with and without any fracture. Patients with any fracture significantly had a longer duration of type 2 diabetes mellitus, (p<0.001), which is similar to a meta-analysis by Wang et al. [30], which showed that the pooled relative risk for the occurrence of vertebral fracture in those with diabetes compared to people without the disease was 2.03 (95 % CI 1.60-2.59; p < 0.0001). This corresponds to a statistically significant positive association between diabetes and risk of vertebral fracture.

In our study, univariate regression analysis for predictors of vertebral fractures highlighted that significant predictors were amenorrhea (p=0.005) and metrorrhagia (p=0.044), while the multivariate regression analysis demonstrated that the still significant predictors were menopause age ≤ 49 (p=0.020) and amenorrhea (p=0.022). These findings were comparable to a major study conducted in Korea that involved 189,883 participants, including 72,732 participants with vertebral fractures, where vertebral fractures showed statistical significance for age at menarche > 17 years and age at menopause < 55 years, with p-values of 0.001 for both [31].

Extensive research related to our study has shown a link between menstrual history and bone mass later in life. Women with menstrual irregularities may have different plasma estrogen levels; however, they consistently show low plasma levels of progesterone hormone. Although no literature has examined these factors in relation to vertebral fractures, much research has examined these factors in relation to osteoporosis and osteoporotic fractures in general [32].

The study by Christiansen [33] found that women with a history of amenorrhea had lower bone density than did women who consistently had regular menstrual cycles, which was consistent with our study. However, some studies have not found a relationship between menstrual history and postmenopausal bone mass [34-35]; for example, Fox et al. [34] found no differences in bone density in elderly women (65 years and older) with and without a history of regular menstrual cycles.

These results were also consistent in the study by Nguyen et al. [36], where it was reported that younger age at menarche, longer total span of reproductive years, later age at menopause, and shorter menstrual cycle duration are associated with higher bone density in postmenopausal women, whereas premenopausal amenorrhea (cessation of menstruation) or oligomenorrhea (infrequent menstruation) are associated with lower bone density in postmenopausal women and increased risk of lumbar spine fracture (p<0.001).

During our study, menstrual history showed statistical significance between the two groups in terms of menarche age (p=0.010), menopause age (p=0.011), amenorrhea (p=0.003), and metrorrhagia (p=0.035). Our study results were comparable to the study by Shimizu et al. [37], in which during the 10-year period, 250 women reported first onset of vertebral fractures. Women with menarche at ≥16 years and irregular menstruation had a higher risk of developing vertebral fractures when compared to women with age at menarche ≤13 years and regular menstruation (p=0.025) [37]. Also, similar results were reported in the study by Sharmai et al. [38] including 796 women, where 18.5% were identified as having osteoporosis; this cross-sectional survey was conducted in the north of Iran to identify the prevalence of osteoporosis and related risk factors in 2004, where significant correlations were found between osteoporosis, and parameters of age, age at menarche, parity, years of menstruation, educational level, job, physical activity, exercise, BMI, and age at menopause (p=0.001) in all of the mentioned variables [38].

Antiepileptic drugs are indicated as prophylaxis or treatment of seizures; in our study, the comparison between participants regarding the intake of antiepileptics showed no statistical significance (p=0.56). These results were comparable to the study by Heo et al. [39], where no significant differences in BMD or increased osteoporotic fractures including vertebral fractures were found between patients on antiepileptics therapy and healthy controls (p=0.149). Also, the study by Sheth and Hermann [40] showed that monotherapy antiepileptics had no effect on BMD with no statistical significance (p=0.9). However, contrasting results from many studies where antiepileptic use was also found to be significantly more frequently associated with osteoporotic fracture [41-44] and reported higher risk of osteoporotic fracture including vertebral fracture with the use of antiepileptic drugs.

The study has its limitations in that the data collection from a single center, but Ain Shams University is considered as one of the leading tertiary health service providers in Egypt. The second limitation was the small number of recruits in the study, and this was due to the COVID restrictions, where only emergency cases were allowed in the orthopedics department. Despite these limitations, the study highlights the possible risk factors of fragility fracture among Egyptian females as a starting point for future research in this field.

## Conclusions

The study was conducted to identify the prevalence and associated risk factors for osteoporotic fractures in which the participants were divided into two groups: the first was the group with concomitant vertebral fractures (patient group), and the second group was the control group. The included patients' group showed statistical significance between the two study groups, where older age at menarche, younger age at menopause, and history of amenorrhea and metrorrhagia were more prevalent in the group with silent vertebral fracture and can be identified as possible risk factors for vertebral fractures. These results highlight the importance of identifying menstrual history and associated irregularities as possible risk factors for fragility fractures including hidden vertebral fractures. Furthermore, menstrual irregularities can warrant the early investigations and possible treatment of osteoporosis and prevent future fractures or disability.
